# Mechanisms of Neuronal Protection against Excitotoxicity, Endoplasmic Reticulum Stress, and Mitochondrial Dysfunction in Stroke and Neurodegenerative Diseases

**DOI:** 10.1155/2015/964518

**Published:** 2015-10-20

**Authors:** Howard Prentice, Jigar Pravinchandra Modi, Jang-Yen Wu

**Affiliations:** ^1^College of Medicine, Florida Atlantic University, Boca Raton, FL 33431, USA; ^2^Program in Integrative Biology, Florida Atlantic University, Boca Raton, FL 33431, USA; ^3^Center for Complex Systems and Brain Sciences, Florida Atlantic University, Boca Raton, FL 33431, USA; ^4^China Medical University Hospital, Taichung, Taiwan

## Abstract

In stroke and neurodegenerative disease, neuronal excitotoxicity, caused by increased extracellular glutamate levels, is known to result in calcium overload and mitochondrial dysfunction. Mitochondrial deficits may involve a deficiency in energy supply as well as generation of high levels of oxidants which are key contributors to neuronal cell death through necrotic and apoptotic mechanisms. Excessive glutamate receptor stimulation also results in increased nitric oxide generation which can be detrimental to cells as nitric oxide interacts with superoxide to form the toxic molecule peroxynitrite. High level oxidant production elicits neuronal apoptosis through the actions of proapoptotic Bcl-2 family members resulting in mitochondrial permeability transition pore opening. In addition to apoptotic responses to severe stress, accumulation of misfolded proteins and high levels of oxidants can elicit endoplasmic reticulum (ER) stress pathways which may also contribute to induction of apoptosis. Two categories of therapeutics are discussed that impact major pro-death events that include induction of oxidants, calcium overload, and ER stress. The first category of therapeutic agent includes the amino acid taurine which prevents calcium overload and is also capable of preventing ER stress by inhibiting specific ER stress pathways. The second category involves N-methyl-D-aspartate receptor (NMDA receptor) partial antagonists illustrated by S-Methyl-N, N-diethyldithiocarbamate sulfoxide (DETC-MeSO), and memantine. DETC-MeSO is protective through preventing excitotoxicity and calcium overload and by blocking specific ER stress pathways. Another NMDA receptor partial antagonist is memantine which prevents excessive glutamate excitation but also remarkably allows maintenance of physiological neurotransmission. Targeting of these major sites of neuronal damage using pharmacological agents is discussed in terms of potential therapeutic approaches for neurological disorders.

## 1. Introduction

Neuronal excitotoxicity that culminates in neuronal death is a hallmark of cellular responses to major stresses such as those that occur in hypoxia/ischemia injury and in neurodegenerative diseases including Alzheimer's disease (AD), Huntington's disease (HD), and Parkinson's disease (PD). Excitotoxicity arises from a massive release of the neurotransmitter glutamate. Under conditions of cerebral hypoxia and/or ischemia that are characteristic of ischemic stroke, diminished oxygen and glucose availability elicit increased neuronal glutamate release which in turn causes overexcitation of neurons postsynaptically. This high level excitation is known to trigger a cascade of prodeath processes. Glutamate excitotoxicity is associated with a failure to maintain calcium homeostasis in the cell, mitochondrial dysfunction, high level generation of oxidants including reactive oxygen species (ROS) and reactive nitrogen species (RNS), and a loss of mitochondrial membrane potential. Decreased ATP levels, resulting from mitochondrial damage, can contribute to increased levels of oxidants, as can the activation of NADPH oxidase and xanthine oxidase. With severe stress, collapse of the mitochondrial membrane potential may be irreversible, under which circumstances mitochondrial permeability transition pore (MPTP) opening may occur, resulting in apoptosis. In addition to necrosis, which is catastrophic cell death associated with energy loss, other key pathways of cell death signaling include apoptosis, initiated by Bcl-2 family members and MPTP opening, as well as another key prodeath process, namely, ER stress. In the current review article we will examine the major steps that contribute to the induction of cell death through stress from excitotoxicity and hypoxia/ischemia and excessive production of oxidants and we will highlight two categories of neuroprotective agent that are effective in impacting or interrupting important aspects of prodeath cascades. The first category involves the amino acid taurine which acts to restore calcium homeostasis and inhibits two out of the three primary ER stress pathways. The second category of agent is illustrated by two examples of NMDA receptor partial antagonists: (1) S-methyl-N,N-diethyldithiocarbamate sulfoxide (DETC-MeSO) which was shown to protect* in vivo* against infarction that results from transient brain ischemia through inhibiting a subset of endoplasmic reticulum stress (ER stress) pathways and (2) memantine that blocks glutamate receptor mediated calcium influx while in large part maintaining physiological glutamate neurotransmission.

## 2. Neuronal Excitotoxicity

Under conditions of hypoxia/ischemia and in neurodegenerative disorders such as Parkinson's disease or Alzheimer's disease, neuronal cells are subjected to overwhelming ionic and biochemical stresses that induce mitochondrial dysfunction as well as elicit cell death processes. Glutamate is the principal excitatory neurotransmitter in the mammalian nervous system and excessive release of glutamate is a key characteristic of these diseases. Importantly, the excessive quantities of extracellular glutamate are toxic and result in neuronal death. High extracellular glutamate results in activation of N-methyl-D-aspartate (NMDA) receptor and *α*-amino-3-hydroxy-5-methyl-4-isoxazolepropionic acid (AMPA) ionotropic glutamate receptors as well as metabotropic glutamate receptors [1]. Glutamate receptor activation contributes to calcium overload, which in turn activates calcium dependent enzymes, increasing reactive oxygen species and reactive nitrogen species and triggering cascades of cell death. In glutamate excitotoxicity calcium overload and collapse of the mitochondrial membrane potential are two key steps in the commitment of cells to die [2].

Following cerebral ischemia and restoration of the blood supply, prooxidant enzymes and mitochondria generate large quantities of oxidants. Superoxide, a major damaging oxidant, is generated in the mitochondria. Prooxidant enzymes, including xanthine oxidase and NADPH oxidase (NOX), also contribute to generating superoxide. Recent studies on oxygen-glucose deprivation (OGD) treated neurons demonstrate three mechanisms for generating damaging ROS with specific stages of involvement during hypoxia and reoxygenation [3]. The mechanisms were the following: in hypoxia both mitochondria and xanthine oxidase were responsible for ROS generation but during reoxygenation the key source of ROS was NOX.

## 3. The Role of Oxidants in Signaling and Cell Damage

### 3.1. Reactive Oxygen Species

Neurons are vulnerable to toxicity from free radicals, in part because of the limited capabilities of their antioxidant mechanisms [4, 5]. Redox homeostasis in normal neurons involves sustaining intracellular signaling with low levels of ROS and a balanced compatible level of antioxidant mechanisms. Under these circumstances, ROS contribute to cell signaling through several mechanisms, such as modulation of activities of protein kinase pathways including receptor tyrosine kinases, protein kinase C (PKC), and mitogen activated protein kinases (MAP kinases) and through activation of key transcription factors including activator protein-1 (AP-1) and nuclear factor- (NF-) kappa-B [6, 7]. However, in stroke and neurodegenerative disease, high levels of ROS are damaging to macromolecules and elicit prodeath processes.

Dysfunctional mitochondria have been shown to act as an important source of free radicals in neurodegenerative diseases including stroke, AD, and PD. The mitochondrial defects may involve deficiency in energy metabolism and, depending on the disease, are related to defects in complexes I and III (stroke), complex I (Parkinson's disease), complex II (Huntington's disease) [8], or complex IV (Alzheimer's disease) [9] of the electron transport chain (ETC). Deficiencies in energy supply are particularly damaging to neurons, which have a high energy demand and hence mitochondrial dysfunction may contribute to neuronal cell death by either necrosis or apoptosis.

A second major source of ROS in neurons was shown to be NADPH oxidase and recent data on N-methyl-D-aspartate receptor (NMDA receptor) activation in neuronal cultures points to NADPH oxidase as a greater contributor to superoxide elevation following excessive glutamate exposure than the mitochondrion [10]. Importantly, it was recently demonstrated that an NMDA receptor activated production of superoxide can be inhibited by blocking Na/H exchange and eliciting mild acidosis, [11] therefore providing a metabolic mechanism for the link between NMDA receptor activation and superoxide production.

### 3.2. Reactive Nitrogen Species: RNS

Upon NMDA receptor activation, NO is generated from neuronal nitric oxide synthase (nNOS) which is linked to the NMDA receptor through shared interaction with postsynaptic density-95 (PSD-95) [12, 13]. Calcium influx through activated NMDA receptors will activate nNOS and produce NO [14].

Nitric oxide plays an important role in excitotoxicity and under conditions of excessive glutamate receptor activation NO interacts with O_2_^−^ (superoxide) to form the toxic molecule peroxynitrite (ONOO^−^) [6]. Peroxynitrite interferes with mitochondrial respiration and then, through releasing zinc from intracellular stores, it elicits additional mitochondrial damage. NO has also been shown to elicit cell death responses through mechanisms that involve S-nitrosylation of protein targets, where a NO group forms a covalent interaction with a reactive cysteine. Targets of this process that are S-nitrosylated include caspases and metalloproteases [5, 15].

## 4. Calcium Overload and Excitotoxicity

Several channels and transporters known to be activated in ischemia are responsible for altering calcium levels in the cytoplasm and these have been shown to include Na^+^/Calcium exchanger [16], acid sensing ion channels [17], volume regulated ion channels [18, 19], and TRP channels [5, 20]. An inhibition of calcium efflux through the Na^+^/Calcium exchanger during ischemia results in increased calcium accumulation [21].

## 5. Endogenous Antioxidants and the Protective Effects of Ischemic Preconditioning

Oxidant levels in the cell are generally maintained at low levels by endogenous antioxidants including SOD, glutathione peroxidase, and catalase. Other cellular antioxidants, including glutathione, ascorbate, and vitamin E, also contribute to maintaining a low level of oxidants in the cell [6]. The mitochondria have been shown to contain a range of antioxidant systems for coping with elevated ROS and these include ascorbate, mnSOD, catalase, glutathione, glutathione peroxidase, glutathione reductase, and thioredoxin [22, 23].

Reactive oxygen species are known to have the potential to elicit either detrimental or protective effects in the cell. Whereas high levels of ROS tend to be toxic, low levels of ROS production may be important for signaling and these can serve a beneficial role, which is demonstrated in activation of protective pathways during ischemic preconditioning [23, 24]. An important mechanism of ischemic preconditioning in brain is through induction of cellular antioxidant defenses in part through transcriptional activation by nuclear factor erythroid-2-related factor 2 (Nrf-2) of a number of endogenous antioxidant genes [25]. Nrf-2 is a transcription factor that binds to the promoter domain of key genes that include NAD(P)H quinone oxidoreductases (NQO1 and NQO2), glutathione S-transferase, and heme-oxygenase. Nrf-2 activation is mediated by induction of MAPK signaling, through increased ROS as well as by NO induced S-nitrosylation of PKC [26, 27].

Further important pathways of antioxidant induction in preconditioning include those regulated by hypoxia-inducible factor-1 (HIF-1) [28] and by SIRT1 [29]. HIF-1 contributes to cellular survival through activating transcription of a range of protective molecules including heme oxygenase (HO-1) and Bcl-2/adenovirus E1B 19 kDa-interacting protein (BNIP3). BNIP3 has been recently shown to contribute to autophagy and to decrease mitochondrial ROS production [27, 30]. SIRT1 is known to contribute to regulation of expression of antioxidants, which include manganese superoxide dismutase (MnSOD), glutathione peroxidase 1, and catalase.

## 6. Two Major Prodeath Mechanisms: Apoptosis and ER Stress

Under conditions of hypoxia/ischemia, neuronal apoptotic processes are initiated through the actions of proapoptotic Bcl-2 family members including Bax and Bak, to open the mitochondrial permeability transition pore (MPTP) and enable the release of cytochrome C from the mitochondrion into the cytoplasm. Cytochrome C interacts with apoptotic protease activating factor-1 (Apaf-1) to form the apoptosome and caspase 9 becomes activated and initiates a downstream caspase cascade [31]. These caspases cleave several different substrates that include poly (ADP-ribose) polymerase-1 (PARP-1) leading to DNA damage. Furthermore, overactivation of PARP-1 will decrease NADH and ATP which results in energy failure and cellular necrosis [1, 3, 32].

A major site for folding and processing of newly synthesized proteins is the endoplasmic reticulum (ER), which plays a central role in cellular calcium storage and signaling [33]. The initiation and progression of protein processing functions in the ER have been shown to be strictly calcium-requiring processes [34, 35]. When these functions are impaired (a pathological state termed ER stress), unfolded proteins then accumulate in the ER. This protein accumulation represents a severe form of stress that can result in apoptosis if ER function cannot be restored.

Impairment of ER function can arise from depletion of ER calcium stores, oxidative stress, from blocking of the proteasome for degrading unfolded proteins, or from proteins that arise from genetic mutations and cannot be correctly folded. Perturbations in protein folding lead to accumulation of defective proteins in the ER lumen which then represent a signal that is detected by ER sensors eliciting downstream signaling events. ER stress activates the unfolded protein response (UPR), a complex signal-transduction pathway responsible for cellular adaptation to reestablish ER homeostasis. However, under conditions of chronic ER stress, the UPR will elicit cell apoptosis [36]. ER stress is known to play a crucial role in hypoxia/ischemia-induced cell damage [33, 37–39]. The accumulation of misfolded proteins in neurons is associated with excitotoxicity and is found in stroke in addition to a number of neurodegenerative diseases including Alzheimer's disease (AD), amyotrophic lateral sclerosis (ALS), Huntington's disease (HD), and Parkinson's disease (PD).

## 7. Unfolded Protein Response (UPR) Pathways

The UPR serves primarily to restore ER function by decreasing the quantity of misfolded proteins that must be correctly processed in the ER and by enhancing ER protein processing capacity. The UPR is triggered by activation of the following three stress sensors on the ER membrane: double-stranded RNA-activated protein kinase-like ER kinase (PERK), activating transcription factor 6 (ATF6), and inositol-requiring kinase 1a (IRE1a). The sensing mechanism is understood to involve the ER chaperone Grp78/Bip which recognizes unfolded proteins and then dissociates from each of the three sensing molecules, releasing them from inhibition [36].

In neurons, under conditions of physiological homeostasis, PERK, ATF6, and IRE1a interact with Grp78, but when the ER is dysfunctional, Grp78 dissociates resulting in phosphorylation of PERK and IRE1a and cleavage of ATF6 (P90) to ATF6 (P50) [40]. Activated PERK has been shown to phosphorylate eukaryotic initiation factor 2a (eIF2a) which induces suppression of global protein synthesis while also increasing translation of activating transcription factor 4 (ATF-4) [36]. Under activated conditions, the second sensor IRE1a signals to regulate the mRNA for the transcription factor X box binding protein (XBP1). XBP1 is responsible for regulating a specific subset of UPR target genes, involved in folding and ER-associated degradation (ERAD) [41]. A second function of IRE1a is to bind to adaptor proteins in the cytosol and activate signaling pathways known as alarm pathways (including JNK, Ask1, and NF-kappaB) resulting in activation of autophagy and apoptosis [36]. The third stress sensor, ATF6, a membrane spanning protein, dissociates from Grp78 and then becomes proteolytically cleaved before translocating to the nucleus to contribute to induction of protein quality control genes [33].

Under conditions of severe stress, following PERK activation and subsequent eIF2a phosphorylation, elevated ATF4 contributes to cell death processes by upregulating transcription of proapoptotic Bcl-2 family members in addition to the key transcription factor C/EBP homologous protein (CHOP), which controls transcription of genes encoding both pro- and antiapoptotic Bcl-2 family members. All of these three stress sensing pathways upregulate the transcription factor CHOP and are therefore capable of contributing to cell fate decisions, by altering levels of Bcl-2 family members to elicit apoptosis [42]. A further specialized caspase mechanism involves caspase-12, which is an ER membrane-associated caspase that is upregulated by glutamate excitotoxicity and then activates the caspase pathway cascade hence transducing prodeath signals and apoptosis [43].

## 8. Therapeutic Interventions Targeting Prodeath Mechanisms

### 8.1. Taurine Induced Protection through Mechanisms Involving Inhibition of Excitotoxicity, Calcium Overload, Oxidative Stress, and ER Stress

Taurine is the most abundant amino acid in brain, skeletal muscle, and cardiac muscle and has been investigated as a potential therapeutic agent in experimental models of several neurodegenerative diseases including stroke, Alzheimer's disease, and Huntington's disease. Taurine is responsible for contributing to several different cellular processes including neuromodulation, neurotransmission, regulation of calcium dependent functions, acting as an osmolyte, and maintaining the structural integrity of the membrane [44, 45]. Taurine can also serve as a neuroprotective agent to combat glutamate toxicity and H_2_O_2_ induced cell injury [45]. Previously, it was demonstrated that taurine protected against glutamate induced increases in intracellular free calcium. It was subsequently shown that taurine inhibited glutamate induced calcium influx through L-, P/Q, and N-type voltage gated calcium channels and the NMDA receptor channel [46].

Although taurine is known to be protective against oxidative stress, in a number of tissues it was demonstrated by Aruoma et al. (1988) [47] that taurine is not able to directly scavenge reactive oxygen species. Taurine may be able to restore endogenous antioxidant levels in stressed cells and this effect has been demonstrated in several cell types including neurons, vascular smooth muscle cells, and liver. Taurine has also been found to upregulate antioxidant defenses in normal cells, as shown in a study by Vohra and Hui (2001) [48], demonstrating increased superoxide dismutase and glutathione peroxidase in unstressed neurons. Taurine may block generation of free radicals, through inhibiting cytoplasmic calcium increases, and as a consequence preventing mitochondrial dysfunction [49]. Another recently described antioxidant effect of taurine involves a role of this key amino acid in a mechanism underlying the correct translation and expression of mitochondrial proteins. A deficiency in this function is found in the disease known as mitochondrial myopathy, encephalopathy, lactic acidosis, and stroke-like episodes (MELAS), which is caused by a specific mutation in a particular taurine conjugated tRNA. The need for taurine is central to the nature of this disorder because the mutated mitochondrial tRNA leu (UUR) fails to modify uridine to 5-taurinomethyluridine. Consequently, defective translation of mitochondrial encoded proteins ensues, eliciting ETC dysfunction and superoxide generation [50, 51]. MELAS presents as a cluster of clinical symptoms that include neuropathy, myopathy, cardiomyopathy, endocrine modifications and retinopathy.

In examining the effects of taurine on primary neuronal cultures, it was found that taurine protected against glutamate excitotoxicity and against cellular damage, following hypoxia/reoxygenation, through regulation of key ER stress pathways. Specifically taurine suppressed the upregulation of caspase-12 and CHOP, following reoxygenation, pointing to a significant involvement in combating ER stress [40]. In a detailed analysis of the three major ER stress pathways, it was found that taurine could downregulate the ratio of cleaved ATF-6 to full length ATF-6 and decrease expression of p-IRE1. Taurine protected against ER stress, following either hypoxia/reoxygenation or glutamate treatment, through suppressing ATF6 and IRE1 pathways but in these studies taurine had no detectable effect on PERK pathway activation [40].

### 8.2. Partial NMDA Receptor Antagonists

#### 8.2.1. DETC-MeSO

Partial blocking of glutamate receptors shows considerable promise in strategies to combat stroke and neurodegenerative disease. S-Methyl-N,N-diethyldithiocarbamate sulfoxide (DETC-MeSO), a metabolite of disulfiram, which has been used to treat alcoholism for more than 5 decades, is known to be a partial NMDA receptor antagonist. DETC-MeSO selectively and specifically blocks NMDA receptors and was shown to be protective against glutamate excitotoxicity in primary rat neuronal cultures.

In* in vivo* mouse studies DETC-MeSO pretreatment prevented ethanol induced kindling seizures, as well as seizures induced by either NMDA or ammonium acetate, all of which are mediated by NMDA receptors [52]. Using a rat model of transient focal cerebral ischemia, the effects of DETC-MeSO were examined on infarct size as well as on specific ER stress pathways. DETC-MeSO was found to provide potent neuroprotection, by reversing the ischemia induced activation of the PERK pathway components, in both the core and the penumbra [53]. The results also implicated inhibition of downstream components of the IRE-1 pathway in this neuroprotection. By contrast the ATF-6 pathway of ER stress was not activated in response to DETC-MeSO treatment.

#### 8.2.2. Memantine

Studies on the partial NMDA receptor antagonist memantine indicate that this drug is capable of blocking pathological NMDA receptor pathways, while maintaining physiological functions. Memantine has a low affinity for the NMDA receptor although it has selectivity in terms of its action on the NMDA receptor. The “off-rate” of a drug is intrinsic to the nature of the drug-receptor complex and memantine's low affinity for the NMDA receptor arises, because of the drug's fast off-rate [54]. Memantine inhibits by blocking the NMDA receptor associated ion channel when excessive channel opening occurs. Memantine falls into the category of an uncompetitive antagonist in that its action is dependent upon previous activation of the receptor by the agonist. As a result, a set concentration of antagonist blocks a high concentration of agonist better than it blocks a low concentration of agonist [54]. In combination, the uncompetitive inhibitor mechanism, together with its fast off-rate, enables memantine to block excessively opened NMDA receptor channels, while mostly sparing physiological neurotransmission. In a rat stroke model memantine delivered after the ischemic insult substantially decreased the area of infarct [55, 56].

Memantine is currently an approved drug in Europe and the USA for treatment of moderate-to-severe Alzheimer's disease. Beta-amyloid as soluble oligomers is proposed as the major cause of synaptic dysfunction in Alzheimer's disease. Soluble oligomeric beta-amyloid is known to interact with proteins, which contribute to maintaining glutamate homeostasis, and notably the NMDA receptor. Recent evidence indicates that increased cytosolic calcium, induced by beta-amyloid in neuronal cultures, was only slightly decreased by ifenprodil, an antagonist to NMDA receptors containing the NRB2B subunit. The data suggested that beta-amyloid oligomers directly activate NR2A subunit containing NMDA receptors [57]. Interestingly, in addition to its antagonist function on NMDA receptors, memantine has been reported to decrease levels of secreted APP, A-Beta (1–40), and A-Beta (1–42) as well as decreasing secretion of A-Beta (1–42) in neuroblastoma cells and neuronal cultures [58, 59].

### 8.3. Concluding Remarks

Included in the initiating stimuli for neuronal prodeath pathways are glutamate excitotoxicity, calcium overload, and high level oxidant production. In this paper we have described classes of therapeutic agent, which may contribute towards preventing these early events, including taurine and the partial NMDA antagonists DETC-MeSO and memantine. Major sites of neuronal damage and potential therapeutic agents indicating their sites of action are presented in Table 1. Glutamate toxicity, calcium overload, and increased oxidants may activate downstream pathways such as apoptosis and the three distinct ER stress pathways. A number of drugs, including taurine and DETC-MeSO, have been shown to inhibit apoptosis cascades and specific subsets of the major ER stress pathways. Current and ongoing therapeutic strategies for stroke and neurodegenerative disease are likely to incorporate targeting of specific signaling mechanisms and to combat these, either in the early adaptations to severe stress just mentioned or during the downstream signaling events, such as in apoptotic caspase activation or in ER stress signaling (such as PERK, ATF6, IRE-1, or CHOP activation). An understanding of the specific prodeath components that can be successfully blocked should aid with finding both the optimal timing for the protective actions of therapeutic agents and the most appropriate choice of drugs for particular diseases, such as stroke, Alzheimer's disease, or other neurodegenerative disorders.

## Figures and Tables

**Table 1:** Major sites of neuronal damage and potential therapeutic agents indicating their sites of neuroprotective action.

Important sites for protection in neuronal stress		
	Type of neuronal stress	Neuroprotective agent
1	Excitotoxicity	DETC-MeSO, memantine, or taurine
2	Calcium overload	Taurine or preconditioning mechanisms
3	Increases in oxidative stress	Taurine or preconditioning mechanisms
4	Apoptosis	Taurine, DETC-MeSO, or preconditioning mechanisms
5	Endoplasmic reticulum stress	Taurine or DETC-MeSO

## References

[1] Hara M. R., Snyder S. H. (2007). Cell signaling and neuronal death. *Annual Review of Pharmacology and Toxicology*.

[2] Abramov A. Y., Duchen M. R. (2008). Mechanisms underlying the loss of mitochondrial membrane potential in glutamate excitotoxicity. *Biochimica et Biophysica Acta*.

[3] Abramov A. Y., Scorziello A., Duchen M. R. (2007). Three distinct mechanisms generate oxygen free radicals in neurons and contribute to cell death during anoxia and reoxygenation. *Journal of Neuroscience*.

[4] Adibhatla R. M., Hatcher J. F. (2010). Lipid oxidation and peroxidation in CNS Health and disease: from molecular mechanisms to therapeutic opportunities. *Antioxidants and Redox Signaling*.

[5] Moskowitz M. A., Lo E. H., Iadecola C. (2010). The science of stroke: mechanisms in search of treatments. *Neuron*.

[6] Chen H., Yoshioka H., Kim G. S. (2011). Oxidative stress in ischemic brain damage: mechanisms of cell death and potential molecular targets for neuroprotection. *Antioxidants & Redox Signaling*.

[7] Dröge W. (2002). Free radicals in the physiological control of cell function. *Physiological Reviews*.

[8] Damiano M., Galvan L., Déglon N., Brouillet E. (2010). Mitochondria in Huntington's disease. *Biochimica et Biophysica Acta*.

[9] Alleyne T., Mohan N., Joseph J., Adogwa A. (2011). Unraveling the role of metal ions and low catalytic activity of cytochrome C oxidase in Alzheimer's disease. *Journal of Molecular Neuroscience*.

[10] Brennan A. M., Suh S. W., Won S. J. (2009). NADPH oxidase is the primary source of superoxide induced by NMDA receptor activation. *Nature Neuroscience*.

[11] Lam T. I., Brennan-Minnella A. M., Won S. J. (2013). Intracellular pH reduction prevents excitotoxic and ischemic neuronal death by inhibiting NADPH oxidase. *Proceedings of the National Academy of Sciences of the United States of America*.

[12] Brenman J. E., Chao D. S., Gee S. H. (1996). Interaction of nitric oxide synthase with the postsynaptic density protein PSD-95 and *α*1-syntrophin mediated by PDZ domains. *Cell*.

[13] Nakamura T., Tu S., Akhtar M., Sunico C., Okamoto S.-I., Lipton S. (2013). Aberrant Protein S-nitrosylation in neurodegenerative diseases. *Neuron*.

[14] Bredt D. S., Hwang P. M., Glatt C. E., Lowenstein C., Reed R. R., Snyder S. H. (1991). Cloned and expressed nitric oxide synthase structurally resembles cytochrome P-450 reductase. *Nature*.

[15] Gu Z., Kaul M., Yan B. (2002). S-nitrosylation of matrix metalloproteinases: signaling pathway to neuronal cell death. *Science*.

[16] Bano D., Nicotera P. (2007). Ca^2+^ signals and neuronal death in brain ischemia. *Stroke*.

[17] Xiong Z.-G., Zhu X.-M., Chu X.-P. (2004). Neuroprotection in ischemia: blocking calcium-permeable acid-sensing ion channels. *Cell*.

[18] Kimelberg H. K., MacVicar B. A., Sontheimer H. (2006). Anion channels in astrocytes: biophysics, pharmacology, and function. *Glia*.

[19] Simard J. M., Kent T. A., Chen M., Tarasov K. V., Gerzanich V. (2007). Brain oedema in focal ischaemia: molecular pathophysiology and theoretical implications. *Lancet Neurology*.

[20] Aarts M. M., Tymianski M. (2005). TRPMs and neuronal cell death. *Pflugers Archiv European Journal of Physiology*.

[21] Abe T., Kunz A., Shimamura M., Zhou P., Anrather J., Iadecola C. (2009). The neuroprotective effect of prostaglandin E2 EP1 receptor inhibition has a wide therapeutic window, is sustained in time and is not sexually dimorphic. *Journal of Cerebral Blood Flow & Metabolism*.

[22] Andreyev A. Y., Kushnareva Y. E., Starkov A. A. (2005). Mitochondrial metabolism of reactive oxygen species. *Biochemistry*.

[23] Perez-Pinzon M. A., Stetler R. A., Fiskum G. (2012). Novel mitochondrial targets for neuroprotection. *Journal of Cerebral Blood Flow and Metabolism*.

[24] Ambrosio G., Tritto I., Chiariello M. (1995). The role of oxygen free radicals in preconditioning. *Journal of Molecular and Cellular Cardiology*.

[25] de Vries H. E., Witte M., Hondius D. (2008). Nrf2-induced antioxidant protection: a promising target to counteract ROS-mediated damage in neurodegenerative disease?. *Free Radical Biology and Medicine*.

[26] Kaspar J. W., Niture S. K., Jaiswal A. K. (2012). Antioxidant-induced INrf2 (Keap1) tyrosine 85 phosphorylation controls the nuclear export and degradation of the INrf2-Cul3-Rbx1 complex to allow normal Nrf2 activation and repression. *Journal of Cell Science*.

[27] Thompson J. W., Narayanan S. V., Perez-Pinzon M. A. (2012). Redox signaling pathways involved in neuronal ischemic preconditioning. *Current Neuropharmacology*.

[28] Ara J., Fekete S., Frank M., Golden J. A., Pleasure D., Valencia I. (2011). Hypoxic-preconditioning induces neuroprotection against hypoxia-ischemia in newborn piglet brain. *Neurobiology of Disease*.

[29] Raval A. P., Dave K. R., Pérez-Pinzón M. A. (2006). Resveratrol mimics ischemic preconditioning in the brain. *Journal of Cerebral Blood Flow & Metabolism*.

[30] Zhang H., Bosch-Marce M., Shimoda L. A. (2008). Mitochondrial autophagy is an HIF-1-dependent adaptive metabolic response to hypoxia. *The Journal of Biological Chemistry*.

[31] Yoshida H., Kong Y.-Y., Yoshida R. (1998). Apaf1 is required for mitochondrial pathways of apoptosis and brain development. *Cell*.

[32] Dawson V. L., Dawson T. M. (2004). Deadly conversations: nuclear-mitochondrial cross-talk. *Journal of Bioenergetics and Biomembranes*.

[33] Paschen W., Mengesdorf T. (2005). Endoplasmic reticulum stress response and neurodegeneration. *Cell Calcium*.

[34] Lodish H. F., Kong N., Wikström L. (1992). Calcium is required for folding of newly made subunits of the asialoglycoprotein receptor within the endoplasmic reticulum. *The Journal of Biological Chemistry*.

[35] Kuznetsov G., Brostrom M. A., Brostrom C. O. (1992). Demonstration of a calcium requirement for secretory protein processing and export. Differential effects of calcium and dithiothreitol. *The Journal of Biological Chemistry*.

[36] Mercado G., Valdés P., Hetz C. (2013). An ERcentric view of Parkinson's disease. *Trends in Molecular Medicine*.

[37] Azfer A., Niu J., Rogers L. M., Adamski F. M., Kolattukudy P. E. (2006). Activation of endoplasmic reticulum stress response during the development of ischemic heart disease. *The American Journal of Physiology—Heart and Circulatory Physiology*.

[38] DeGracia D. J., Montie H. L. (2004). Cerebral ischemia and the unfolded protein response. *Journal of Neurochemistry*.

[39] Kumar R., Azam S., Sullivan J. M. (2001). Brain ischemia and reperfusion activates the eukaryotic initiation factor 2*α* kinase, PERK. *Journal of Neurochemistry*.

[40] Pan C., Prentice H., Price A. L., Wu J.-Y. (2012). Beneficial effect of taurine on hypoxia-and glutamate-induced endoplasmic reticulum stress pathways in primary neuronal culture. *Amino Acids*.

[41] Hetz C. (2012). The unfolded protein response: controlling cell fate decisions under ER stress and beyond. *Nature Reviews Molecular Cell Biology*.

[42] Oyadomari S., Mori M. (2004). Roles of CHOP/GADD153 in endoplasmic reticulum stress. *Cell Death and Differentiation*.

[43] Yoneda T., Imaizumi K., Oono K. (2001). Activation of caspase-12, an endoplastic reticulum (ER) resident caspase, through tumor necrosis factor receptor-associated factor 2-dependent mechanism in response to the ER stress. *Journal of Biological Chemistry*.

[44] Menzie J., Pan C., Prentice H., Wu J.-Y. (2014). Taurine and central nervous system disorders. *Amino Acids*.

[45] Wu J.-Y., Prentice H. (2010). Role of taurine in the central nervous system. *Journal of Biomedical Science*.

[46] Wu H., Jin Y., Wei J., Jin H., Sha D., Wu J.-Y. (2005). Mode of action of taurine as a neuroprotector. *Brain Research*.

[47] Aruoma O. I., Halliwell B., Hoey B. M., Butler J. (1988). The antioxidant action of taurine, hypotaurine and their metabolic precursors. *Biochemical Journal*.

[48] Vohra B. P. S., Hui X. (2001). Taurine protects against carbon tetrachloride toxicity in the cultured neurons and in vivo. *Archives of Physiology and Biochemistry*.

[49] El Idrissi A., Trenkner E. (1999). Growth factors and taurine protect against excitotoxicity by stabilizing calcium homeostasis and energy metabolism. *Journal of Neuroscience*.

[50] Schaffer S. W., Azuma J., Mozaffari M. (2009). Role of antioxidant activity of taurine in diabetes. *Canadian Journal of Physiology and Pharmacology*.

[51] Schaffer S. W., Shimada K., Jong C. J., Ito T., Azuma J., Takahashi K. (2014). Effect of taurine and potential interactions with caffeine on cardiovascular function. *Amino Acids*.

[52] Ningaraj N. S., Chen W., Schloss J. V., Faiman M. D., Wu J.-Y. (2001). S-methyl-N,N-diethylthiocarbamate sulfoxide elicits neuroprotective effect against N-methyl-*D*-aspartate receptor-mediated neurotoxicity. *Journal of Biomedical Science*.

[53] Mohammad-Gharibani P., Modi J., Menzie J. (2014). Mode of action of *S*-methyl-*N*, *N*-diethylthiocarbamate sulfoxide (DETC-MeSO) as a novel therapy for stroke in a rat model. *Molecular Neurobiology*.

[54] Gu Z., Nakamura T., Lipton S. A. (2010). Redox reactions induced by nitrosative stress mediate protein misfolding and mitochondrial dysfunction in neurodegenerative diseases. *Molecular Neurobiology*.

[55] Chen H.-S. V., Pellegrini J. W., Aggarwal S. K. (1992). Open-channel block of N-methyl-D-aspartate (NMDA) responses by memantine: Therapeutic advantage against NMDA receptor-mediated neurotoxicity. *Journal of Neuroscience*.

[56] Chen H.-S. V., Wang Y. F., Rayudu P. V. (1998). Neuroprotective concentrations of the N-methyl-D-aspartate open-channel blocker memantine are effective without cytoplasmic vacuolation following post-ischemic administration and do not block maze learning or long-term potentiation. *Neuroscience*.

[57] Texidó L., Martín-Satué M., Alberdi E., Solsona C., Matute C. (2011). Amyloid *β* peptide oligomers directly activate NMDA receptors. *Cell Calcium*.

[58] Danysz W., Parsons C. G. (2012). Alzheimer's disease, *β*-amyloid, glutamate, NMDA receptors and memantine—searching for the connections. *British Journal of Pharmacology*.

[59] Alley G. M., Bailey J. A., Chen D. (2010). Memantine lowers amyloid-beta peptide levels in neuronal cultures and in APP/PS1 transgenic mice. *Journal of Neuroscience Research*.

